# Tobacco use and other aspects related to smoking among school-going adolescents aged 13–15 years in Malaysia: Analysis of three cross-sectional nationally representative surveys in 2003, 2009 and 2016

**DOI:** 10.18332/tid/127231

**Published:** 2020-09-17

**Authors:** Kuang H. Lim, Sumarni M. Ghazali, Hui L. Lim, Chee C. Kee, Yong K. Cheah, Balvinder S. G. Pradhaman Singh, Pei P. Heng, Chien H. Teh, Yoon L. Cheong, Jia H. Lim

**Affiliations:** 1 Special Research Centre, Institute for Medical Research, Kuala Lumpur, Malaysia; 2 Department of Oncology, Hospital Sulltan Ismail, Johor Bahru, Malaysia; 3 Sector for Biostatistics and Data Repository, National Institute of Health, Shah Alam, Malaysia; 4 School of Economics, Finance and Banking, Universiti Utara Malaysia, Sintok, Malaysia; 5 School of Pharmacy, Monash University Malaysia, Subang Jaya, Malaysia

**Keywords:** tobacco use, school-going adolescents, global youth tobacco survey, secondhand smoke

## Abstract

**INTRODUCTION:**

Periodic surveys on tobacco use patterns and other aspects of tobacco use among school-going adolescents in Malaysia provide information on the effectiveness of anti-smoking measures implemented. However, such information is limited in Malaysia. We investigated the prevalence of smoking and other related aspects among middle-secondary school students in Malaysia from the years 2003–2016 to fill this gap.

**METHODS:**

We analyzed data from the Global Youth Tobacco Survey (GYTS) 2003, GYTS 2009, and the Tobacco and Electronic Cigarette Survey among Malaysia Adolescents (TECMA) 2016. The surveys employed multistage sampling to select representative samples of adolescents attending secondary school in Malaysia. Data were collected using a pre-validated self-administered anonymous questionnaire adopted from the GYTS.

**RESULTS:**

Between 2003 and 2016, major changes occurred in which there were reductions in the prevalence of ever smoking, current smoking, and susceptibility to smoking. Reductions were also observed in exposure to SHS in public places and in the home. The proportion of school-going adolescents who support a ban on smoking in public places increased between 2013 to 2016, and there was a significant reduction in the proportion of respondents that were offered ‘free’ cigarettes by tobacco company representatives. However, there was no difference in the proportion of adolescents who initiated smoking before the age of 10 years and current smokers seeking advice to quit smoking across the time period.

**CONCLUSIONS:**

Our study indicates that the smoking policies and measures have been effective in reducing smoking prevalence, secondhand smoke exposure, and access to cigarettes, among school-going adolescents in Malaysia. However, measures to reduce smoking initiation and increase smoking cessation need to be strengthened to reduce the burden of smoking-related diseases in Malaysia in the long-term.

## INTRODUCTION

Tobacco use is one of the most important preventable causes of premature death, disease and disability around the world^1^. Smoking is also a significant cause of health problems in Malaysia2, smoking-related disease having been a major contributor to mortality since 1980^3^, with a third of diseases due to smoking4. Several nationwide studies have reported that more than 70% of smokers started to smoke before the age of 18 years; this is in line with other studies conducted both locally and in other countries^2,4,5^, which revealed that smoking is a learned behavior and usually begins during adolescence. The earlier individuals start smoking, the higher their risk to develop diseases related to smoking^6^ and the less likelihood to cease smoking during their adulthood^7,8^. Therefore, action to prevent non-smoking youth from smoking is among the initiatives included in the policy of the Ministry of Health, so that the problem of smoking-related illness in Malaysia can be addressed^3^.

The Malaysian government, through several ministries, has introduced policies and measures to reduce the incidence of smoking among Malaysian adolescents. These initiatives include health promotion activities such as ‘Young Doctor Programmes’ to teach youth about the health risks of smoking^9^, introduction of the Control of Tobacco Regulation 2004, which includes the prohibition of access to tobacco for people aged <18 years, a ban on the sale of cigarettes in a ‘kiddy-pack’, and the expansion of smoke-free public areas^10^. All policies that were introduced were in-line with the Framework Convention of Tobacco Control^11^, which was ratified by the Malaysian Government in 2005. In addition, the Malaysian government (i.e. Ministry of Finance) has also changed the taxation structure of smoking from the weight to stick of cigarettes, thus increasing the price of cigarettes as a monetary measure to reduce the demand for cigarette products^3^.

The Framework Convention of Tobacco Control (FCTC) suggested the following six MPOWER measures to reduce the demand for tobacco and to monitor the progress of anti-smoking in member countries^11^: Monitor tobacco use and prevention policies; Protect people from tobacco smoke; Offer help to quit tobacco use; Warn about the danger of tobacco; Enforce bans on tobacco advertising, promotion and sponsorship; and Raise taxes for tobacco. However, no due attention was given to the monitoring of smoking among middle-school students aged 13–15 years in Malaysia. Although studies among young people smoking in Malaysia have increased since 2006, most of the studies have concentrated on smoking prevalence and other factors associated with smoking among youth^12,13^. Therefore, we examined the following four MPOWER measures: prevalence of cigarette smoking, secondhand smoke exposure, smoking cessation, access to tobacco and sponsorship, as well as susceptibility to smoking, using data from three national smoking surveys to obtain a clearer picture of the status of tobacco use and control among middle-school adolescents in Malaysia.

## METHODS

### Source of data

Data were analyzed from three national smoking surveys: The Global Youth Tobacco Survey (GYTS) 2003, GYTS 2009, and the Tobacco and Electronic Cigarette Survey among Malaysia Adolescents (TECMA) 2016. The surveys GYTS 2003 and GYTS 2009 used a two-stage cluster sampling design to select a representative sample of lower secondary students aged 13–15 years enrolled in public schools under the Ministry of Education, Malaysia. In the first stage, secondary schools were sampled based on probability of selection equal to school enrolment size; in the second stage, classes within the chosen schools were sampled using systematic equal-probability sampling with random start. All students in the selected classes were invited to participate in the survey. A total of 50 secondary schools were selected for the study. Ethical approval was obtained from the Medical Research Ethical Committee, Ministry of Health Malaysia, and Ethical Committee, Ministry of Education, prior to the study. Written consent was obtained from both the parents/guardians of all selected participants and the participants themselves, prior to the interview session. The Director-General of Health, Malaysia, gave permission for publication of the study. The authors may be contacted for data requests.

In the TECMA survey, cross-sectional and multistage cluster sampling was employed to select a representative sample of school-going adolescents aged 10–19 years. However, for the purpose of this study, only data from respondents aged 13–15 years were selected for analysis. In the TECMA sampling design, the primary strata consisted of states in Malaysia, and the secondary strata were urban and rural areas within the states. The primary sampling units were primary and secondary schools in each state while the secondary sampling units were classes in the selected schools. All students in the selected classes were eligible to participate in the study. In total, 138 schools were selected (82 urban and 56 rural). A full description of the study methodology is available elsewhere^14^. All three surveys utilized the latest sampling frame provided by Ministry of Education, Malaysia.

The questionnaire for GYTS 2003 was adopted from the GYTS Core Questionnaire with Optional Questions^15^. A panel of experts selected the country-specific optional items to be included in the final questionnaire. The final country questionnaire was translated into the local language and back-translated to check for accuracy. GYTS country research coordinators conducted focus groups of students aged 13–15 years to further test the accuracy of the translation and student understanding of the questions. Face validity was established through a pre-test among 30 secondary school students aged 13–15 years. The same questionnaire was used in the GTYS 2009 and TECMA 2016 surveys. The questionnaire assessed smoking prevalence, perceptions and attitudes about tobacco, access to and availability of tobacco products, susceptibility to initiate smoking, exposure to secondhand smoke, school curriculum, media and advertising, and smoking cessation.

### Definition of smokers

**Ever smoker^16^:** Respondents who reported to have smoked at least once in their lifetime.

**Current smoker^17^:** Those who smoked cigarettes on 1 or more days in the past 30 days.

**Never smoker^17^:** A person who had never tried or experimented with cigarette smoking, even one or two puffs.

### Statistical analyses

All statistical analyses were performed using SPSS version 22. A weighting factor was applied to each subject to adjust for non-response (by school, class, and student) and variation in the probability of selection at the school, class, and student levels (for GYTS 2003 and GYTS 2009), whilst for TECMA, the sample was weighted based on the variation in the probability of selection at the state, urban/rural, school, class, and student levels. This was followed by a final adjustment summing up the weights by grade and gender to the population of schoolchildren in the selected grades in each sample site. Differences were considered statistically significant if confidence intervals did not overlap. All analyses were performed at the 95% confidence level using the complex sample analysis.

## RESULTS

### Sociodemographic characteristics

The number of participants in each study were: 3437 for GYTS 2003; 3185 for GYTS 2009; and 5087 for the TECMA 2016. The gender ratio was similar in all three surveys and one-third of the respondents studied in Form 1 (age 12–13 years). There was no significant difference in the distribution of subjects by gender and Form (school grade) between the surveys (Table 1).

**Table 1:** Characteristics of respondents of surveys GYTS 2003, GYTS 2009 and TECMA 2016

*Variable*	*GYTS 2003*			*GYTS 2009*			*TECMA 2016*		
	N*	n**	*% (95% CI)*	N*	n**	*% (95% CI)*	N*	n**	*% (95% CI)*
**Gender**									
Male	592135	1736	50.6 (47.6–53.6)	656798	1640	50.7 (49.0–52.5)	761489	2713	52.7 (50.1–55.3)
Female	578121	1701	49.4 (46.4–52.4)	638140	1582	49.3 (47.0–51.0)	682650	2374	47.3 (44.7–49.9)
**Education level**									
Form 1 (age 12–13)	406861	1267	34.9 (29.3–41.4)	438271	1035	33.9 (32.3–35.6)	478238	1636	33.1 (21.9–46.6)
Form 2 (age 13–14)	402542	1236	327 (28.0–37.7)	426930	974	33.1 (31.4–34.8)	407473	1838	28.2 (20.8–37.1)
Form 3 (age 14–15)	377499	921	32.4 (26.8–38.5)	426072	1205	33.0 (31.4–34.6)	558428	1813	38..7 (27.4–51.3)

* N: estimated population.

** n: sample population.

### Prevalence of smoking

Almost one in three school-going adolescents (27.3%) had ever smoked in 2016 (Table 2) and approximately 1 in 5 ever smokers of both genders had initiated smoking before the age of 10 years. More than 1 in 10 students were current cigarette smokers (14.8%) and almost 10% of never smokers reported likely to initiate smoking in the next year. There was a significant reduction in the proportion of ever smokers, current smokers, and susceptible to smoking, from 2003 to 2009 across gender. The study also found that the proportion of ever, current, and susceptible to smoking, was significantly higher among males compared to females, across the surveys.

**Table 2:** Prevalence of ever smokers, ever smokers who initiated smoking before age of 10 years, current smokers, and non-smokers susceptible to smoking

*Prevalence of smoking*	*Survey*	*Total*		*Male*		*Female*	
		*n*	*% (95% CI)*	*n*	*% (95% CI)*	*n*	*% (95% CI)*
Ever smoked cigarettes, even one or two puffs	TECMA 2016	1258	27.3 (22.5–32.7)	1109	44.9 (40.1–49.8)	149	7.7 (5.2–11.2)
	GYTS 2009	992	31.2 (29.6–32.9)	740	46.9 (44.5–49.4)	203	13.5 (11.8–15.3)
	GYTS 2003	1072	32.6 (31.0–34.3)	887	53.6 (51.5–56.6)	184	11.4 (9.9–13.1)
Ever smokers who initiated smoking before age of 10 years	TECMA 2016	254	22.0 (16.0–29.4)	204	19.7 (14.2–26.2)	50	40.1 (27.7–53.9)
	GYTS 2009	193	22.9 (19.6–25.2)	135	19.5 (16.7–22.6)	58	33.0 (26.4–40.4)
	GYTS 2003	169	16.5 (14.3–18.9)	121	14.1 (11.9–16.7)	48	26.2 (22.5–36.9)
Current cigarette smoker	TECMA 2016	611	14.8 (11.3–19.1)	578	26.1 (19.9–33.4)	33	2.4 (1.4–4.1)
	GYTS 2009	554	18.5 (17.4–19.9)	465	30.9 (29.6–33.3)	80	6.1 (4.9–7.4)
	GYTS 2003	641	19.9 (18.5–21.3)	571	35.5 (33.2–38.0)	70	4.3 (3.4–5.4)
Non-smoker likely to initiate smoking in the next year	TECMA 2016	387	9.6 (7.1–12.9)	252	14.5 (9.5–21.5)	135	5.5 (4.0–7.6)
	GYTS 2009	691	21.7 (20.3–23.2)	516	31.6 (29.6–34.2)	175	11.3 (9.8–13.0)
	GYTS 2003	860	25.8 (24.3–27.4)	674	39.9 (37.6–42.3)	196	11.3 (9.9–13.0)

### Secondhand smoke exposure

More than half of the respondents reported exposure to secondhand smoke (SHS) in public areas (other than the home) (53.8% in 2016 and 63.5% in 2009). Two out of five respondents reported exposure to SHS at home in the past week. The majority (almost 90%) of TECMA 2016 respondents were in favor of a ban on smoking in public places. The prevalence of SHS exposure was significantly lower than GYTS 2009, but the proportion was still high. Support for smoke-free initiatives increased significantly in 2016, where almost 9 in 10 respondents were in favor of the initiatives (Table 3).

**Table 3:** An attempt for smoking cessation among current smokers

*An attempt for smoking cessation*	*Survey*	*Total*		*Male*		*Female*	
		*n*	*% (95% CI)*	*n*	*% (95% CI)*	*n*	*% (95% CI)*
Current cigarette smoker who wants to stop smoking	TECMA 2016	339	74.5 (61.0–84.6)	323	72.3 (57.7–83.5)	16	100
	GYTS 2009	409	75.3 (71.4–78.8)	333	76.9 (72.7–80.6)	76	69.0 (59.6–77.0)
	GYTS 2003	428	79.4 (74.3–83.6)	371	79.2 (73.7–83.8)	57	80.6 (63.5–90.8)
Current cigarette smoker who had tried to stop smoking during the past year	TECMA 2016	352	73.2 (64.5–80.5)	335	71.1 (62.4–78.8)	17	91.5 (83.5–99.7)
	GYTS 2009	465	77.7 (74.1–80.9)	395	80.1 (76.3–83.4)	78	66.9 (57.2–75.4)
	GYTS 2003	550	86.8 (83.1–89.8)	476	86.3 (81.7–89.8)	74	90.5 (83.1–94.9)
Current smoker who had ever received help to stop smoking	TECMA 2016	432	70.2 (66.0–74.1)	412	72.7 (70.2–75.0)	20	42.0 (19.6–69.1)
	GYTS 2009	669	72.5 (69.5–75.3)	531	76.4 (71.0–77.5)	138	66.2 (59.5–72.4)
	GYTS 2003	Not investigated					

### Smoking cessation attempt

Almost 7 in 10 current smokers wanted and attempted to quit smoking (Table 1). The proportion decreased from 79.4% in 2003 to 74.5% in 2016. However, in 2016, all of the smoking female respondents reported contemplating quitting. Seven out of 10 current smokers reported having received assistance to cease smoking, significantly higher in males than in females (Table 4).

**Table 4:** Secondhand smoke exposure at home, in public places, and those who think that smoking should be banned in public places

*Secondhand smoke exposure*	*Survey*	*Total*		*Male*		*Female*	
		*n*	*% (95% CI)*	*n*	*% (95% CI)*	*n*	*% (95% CI)*
Exposed to secondhand smoke at home in the past 7 days	TECMA 2016	1890	40.2 (35.0–45.7)	979	39.9 (35.0–44.9)	911	40.6 (34.1–47.5)
	GYTS 2009	1540	48.5 (46.8–50.3)	799	49.6 (47.2–52.0)	741	47.4 (44.9–49.9)
	GYTS 2003	Not investigated					
Exposed to secondhand smoke in public places in the past 7 days	TECMA 2016	2722	53.8 (50.2–57.5)	1497	56.3 (52.1–61.9)	1225	51.1 (46.9–55.3)
	GYTS 2009	2024	63.5 (61.8–65.2)	1085	66.9 (64.5–69.1)	939	60.1 (57.6–62.5)
	GYTS 2003	Not investigated					
Thinks smoking should be banned in public places	TECMA 2016	4694	87.6 (83.4–90.9)	2344	84.0 (80.3–87.2)	2350	91.7 (85.4–95.4)
	GYTS 2009	2650	83.5 (82.1–84.7)	1273	79.5 (77.4–81.8)	189	87.5 (85.7–89.1)
	GYTS 2003	2731	80.8 (78.1–83.3)	1312	77.4 (73.6–80.7)	1419	84.3 (81.6–86.6)

### Access to tobacco products and health education

About 3 out of every 5 current smokers in 2003 bought their cigarettes, but the proportion was significantly reduced to 1 in 5 in 2016. The proportion of youth who were refused purchase of cigarettes in shops due to being minors increased from 38.9% in 2003 to 54.6% in 2016. A significant reduction was also observed in the proportion of respondents who were ever offered ‘free’ cigarettes by cigarette company representatives (from 14.0% in 2016 to 2.1% in 2016). A majority of the respondents (approximately 80%) were taught about the dangers of tobacco use across the three surveys, and no significant difference was observed between genders (Table 5).

**Table 5:** Access and availability of cigarettes to smokers and Health Education

*Access and availability of cigarettes to smokers and Health Education*	*Survey*	*Total*		*Male*		*Female*	
		*n*	*% (95% CI)*	*n*	*% (95% CI)*	*n*	*% (95% CI)*
Current smoker who usually buys cigarettes in a store	TECMA 2016	221	22.8 (17.2–39.5)	212	25.0 (18.2–33.4)	9	8.0 (2.6–22.5)
	GYTS 2009	309	50.2 (49.4–54.2)	270	53.8 (49.4–58.1)	39	34.6 (26.3–44.0)
	GYTS 2003	393	58.0 (53.8–62.1)	361	61.0 (56.8–65.1)	32	36.9 (27.4–47.6)
Current smoker who usually buys cigarettes in a store and ever denied purchase of cigarettes being a minor	TECMA 2016	182	54.6 (45.2–63.6)	176	57.0 (48.5–65.2)	6	30.8 (9.7–64.8)
	GYTS 2009	267	49.8 (45.5–54.0)	211	49.2 (44.5–53.9)	56	52.0 (42.54–61.4)
	GYTS 2003	134	38.9 (31.8–46.4)	122	38.4 (31.14–46.3)	12	44.0 (23.7–66.0)
Ever offered ‘free’ cigarettes by a cigarette company representative	TECMA 2016	99	2.1 (1.7–2.9)	83	3.6 (2.6–4.8)	16	0.5 (0.3–1.1)
	GYTS 2009	182	5.9 (5.1–6.8)	120	7.5 (6.3–9.0)	62	4.2 (3.3–5.4)
	GYTS 2003	50	14.0 (10.5–18.4)	50	15.3 (10.5–18.4)	-	-
Ever had education in school on dangers of smoking tobacco, in the past year	TECMA 2016	4018	86.4 (81.7–90.1)	2041	85.2 (81.6–88.3)	1977	87.8 (81.4–92.2)
	GYTS 2009	2469	77.7 (76.2–79.2)	1220	74.7 (72.5–76.8)	1269	80.7 (78.7–82.6)
	GYTS 2003	2427	82.0 (78.9–84.7)	1230	81.7 (78.8–84.4)	1197	82.8 (78.5–85.5)

## DISCUSSION

This study is one of the first comprehensive evaluations of the problem of smoking among middle-school students in Malaysia. We found the prevalence of current smokers to be on a downward trend from 2003 to 2016. Similar findings were reported by Miguel-Baquilod et al.^18^ among school-going adolescents in their surveys in 2000 and 2003. However, no similar pattern was observed in two waves of Global Youth Tobacco Survey (GYTS) among school-going adolescents in India^19^ and in Italy^20^. The latest prevalence of 14.8% was similar to the GYTS survey in the Philippines in 2015^21^. However, it is double the rate of 6.7% reported in Bangladesh^22^ as well as 10%, 12.5% and 13.6% from surveys in Thailand^23^, Indonesia^24^, and 68 lower-income and middle-income countries^25^, respectively, although it is 5 percentage points lower than the study reported among youths in Italy^20^. Our study also found that current smoking was significantly higher among males compared to females (ratio 10:1). This is in agreement with several local studies in Indonesia^9,12,13,24^. However, it is in contrast to findings among youths in Italy^20^, which reported a similar proportion of current smokers among boys and girls. We postulate that the results might be due to the local Asian social norm that does not accept female smokers^9^, which has the effect of preventing females from smoking. In addition, the parents and guardians in the local community pay more attention to their daughters, which might be another reason for the findings in this study^9^. The findings suggest that more intervention and anti-smoking measures should target male adolescents in order to reduce the incidence of smoking initiation among them.

Our study also found that approximately 1 in 10 non-smokers intended to initiate smoking in the next year; 16.2% and 12.1% points lower than in 2003 and 2009, respectively. The same finding was also observed in the Philippines, which reported a significant reduction in susceptibility to smoking among youth from 2000 to 2003^18^. However, the findings contradict the increasing trend of susceptibility to smoking among youth in Botswana, which increased from 14.5% in 2001 to 31.5% in 2008^26^, and the recent study of Kamke et al.^27^ among youth in USA, where smoking susceptibility among girls was 20–23% between 1999 and 2011 and increased to 27–29% between 2014 and 2018, whilst the prevalence among boys was 20–23% between 1999 and 2011 and increased to 27–31% between 2014 and 2018. The prevalence of susceptibility to smoking in this study was lower than the 12.5% reported globally^28^. However, the prevalence was similar to the 10.1% reported among school-going adolescents in Thailand^29^. The significant reduction of smoking prevalence from 2003 to 2009 might be due to health promotion and comprehensive legislation implemented since 2005, which created a non-conducive environment for smoking among youth. The prevalence of susceptibility to smoking was significantly higher among males, in line with previous findings^30,31^ and those of Lee et al.^9^ among Thai youth. This may be explained by male smoking having been accepted as a norm in society^9^.

We predict low incidence of future smoking initiation among Malaysian school-going adolescents based on the findings of the present study. However, the existing anti-smoking measures should be strengthened, with focus on early adolescents to further reduce smoking incidence. One in five adolescent smokers started smoking before the age of 10 years, and the proportion has plateaued since 2009, albeit lower than the four out of ten smokers reported among adolescent students in Bangladesh^32^. The findings suggest that more robust and comprehensive measures involving parents or guardians and the school administrators are urgently needed to address the adolescent smoking.

### SHS exposure

The study found a significant reduction in SHS exposure in public areas and at home over the eight-year period. However, the latest prevalence is still high compared to other studies that reported approximately 30.4% and 44.2% of adolescents in 168 lower- and middle-income countries (LMICs)^33^, and about 21% and 39% in 25 African countries^34^, exposed to SHS inside and outside of homes, respectively. However, it is lower than 70.0% (inside the house) and 67.4% (outside the house) reported among youth in Greece^35^, although a decrease in SHS exposure was observed. The prevalence of exposure to SHS remains at levels that will cause major public health problems in the future and does not comply with Article 8 of the World Health Organization (WHO) FCTC, which highlights the importance of protection from exposure to tobacco smoke. It is also well known that SHS has been classified by the International Agency for Research on Cancer as a human carcinogen^36^. Therefore, there is a need to increase the enforcement of existing laws governing smoking and exposure to secondhand smoke in public places. In addition, creating more smoke-free areas and educating the public about the dangers of secondhand smoke will have complementary effects in reducing SHS exposure among people who do not smoke, especially children and adolescents^37^.

Furthermore, health promotion and smoking cessation programmes that target parents/guardians who smoke can be another strategy to reduce the SHS exposure at home. The studies have shown that SHS exposure was high among youth if their parents/guardians smoke^38,39^. With knowledge, the smoking parent/guardian might be more motivated to smoke outside the house^40^; in addition, community intervention recruiting the adults should be intensified to promote smoke-free homes and increase the smoking cessation rate, which will ultimately reduce the SHS exposure at home^41^. Studies revealed that the implementation of a smoke-free home is possible with the consent of parents/guardians, especially in the patriarchal society of Malaysia^9^.

In 2016, only one in five current smokers bought cigarettes from stores themselves, significantly lower than in 2003 (three in five) and in 2009 (50%). The proportion is similar to the 22.6% in Uganda, lower than 26.9% in Ghana, 28.2% in Swaziland, 37.7% in the Republic of Congo, 52.6% in South Africa^42^, and 79.4% in the Philippines^21^. The reduction of almost 30% might be due to a regulation restricting an individual younger than 18 years old to possess cigarettes^3^. The study also revealed that the proportion of smokers who obtained cigarettes was significantly higher among current male smokers across the survey. This might be due to the social norm, whereby smoking among females is disapproved, thus reducing the proportion of female smokers purchasing cigarettes from stores.

Approximately 45.4% of smokers were not refused purchase of cigarettes in 2016, although the proportion is higher compared to those reported in 2003 and 2009. In addition, the prevalence was significantly lower compared to 90% reported by Zulkifli and Rogayah^43^ and among youth in Greece^35^.

The proportion is still substantial, given the smoking regulation, which prohibits the sale of cigarettes to individual younger than 18 years old since 2004^10^. The call for more stringent enforcement activities should be carried out in premises selling tobacco products. As stipulated in the regulation, the study also indicated a need to develop and implement legislation on the access of cigarettes by licensing the premises selling cigarettes, as a substantial proportion of minors are still able to buy cigarettes from stores, as implementation of the rules has been shown to significantly reduce youths’ access to tobacco products^44,45^. Our study also found that only a small proportion of adolescents have ever been offered cigarette products by tobacco industry representatives, 2% lower than a study from six African countries (ranging from 4.7% in Cote d’Ivoire to 12.1% in South Africa)^42^. This finding suggests that continuous efforts should be made to deter this tactic by cigarette companies.

About three-quarters of current smokers wanted to stop smoking and had tried to quit smoking in the past year, across all three surveys, similar to school-going adolescents in Indonesia^24^, but lower than in the Philippines GYTS survey (90%)^21^. The unchanging trend suggests a need to develop, pilottest, and evaluate potential youth cessation programs. Once effective programs have been identified, they need to be implemented throughout the country. A significantly lower proportion of female smokers seek assistance to quit smoking compared to male smokers across the surveys, which is similar to a finding in Indonesia^24^. We postulate that the social norms that stigmatize female smoking hinders female smokers from coming forward to seek assistance to quit smoking.

### Limitations

Several limitations of our study need to be acknowledged. First, there were non-responses in our study that may have introduced selection bias if they were associated with smoking. In addition, the possibility of information or recall bias cannot be excluded given that some students may not provide correct answers and also may under-report the level of smoking, since the data collected are based on a self-administered questionnaire. However, some previous studies have indicated a high correlation between self-reported and biochemically-verified smoking status^46,47^. Second, the data represent only youths who are enrolled in school, which might limit the generalizability to all youths aged 13–15 years in Malaysia. However, the majority of Malaysian adolescents aged 13–15 years attend school as public school education is free^9^. Nevertheless, the surveys achieved a high participation rate, sufficient for drawing firm conclusions, and used a standardized questionnaire, which enables cross-country comparisons. Although the GYTS is cross-sectional, it is a valid and reliable means of tracking changes in the population’s smoking behavior over time and for planning future tobacco control programs.

## CONCLUSIONS

An important finding of the study is the significant reduction in ever and current smoking, intention to smoke within one year, secondhand exposure in public places and homes, access to cigarette products in stores, and offers of ‘free’ cigarette products, among adolescents. However, prevalence of current smoking is still high, and a large proportion of adolescents are still exposed to secondhand smoke and are able to buy tobacco products in stores. The proportion of adolescent smokers who seek assistance to quit and ever taught about the dangers of smoking have also remained stagnant for the past 15 years. The Ministry of Health of Malaysia should involve all stakeholders to formulate and develop a comprehensive tobacco control program to tackle smoking and SHS exposure among children and adolescents, and assess periodically the above measures to evaluate the effectiveness of the implemented programs.
